# Self-touch modulates the somatosensory evoked P100

**DOI:** 10.1007/s00221-015-4355-0

**Published:** 2015-06-24

**Authors:** Hinze Hogendoorn, Marjolein Kammers, Patrick Haggard, Frans Verstraten

**Affiliations:** Experimental Psychology Division, Helmholtz Institute, Universiteit Utrecht, Utrecht, The Netherlands; Institute of Cognitive Neuroscience, University College London, London, UK; Department of Psychology, University of Sydney, Sydney, Australia

**Keywords:** Self-touch, SEPs, Somatosensory evoked potentials, Nociception, Analgesia

## Abstract

It has recently been shown that contact between one’s own limbs (self-touch) reduces the perceived intensity of pain, over and above the well-known modulation of pain by simultaneous colocalized tactile input Kammers et al. (Curr Biol 20:1819–1822, 2010). Here, we investigate how self-touch modulates somatosensory evoked potentials (SEPs) evoked by afferent somatosensory input. We show that the P100 SEP component, which has previously been implicated in the conscious perception of a tactile stimulus, is enhanced during self-touch, as compared to when one is touching nothing, an inanimate object, or another person. A follow-up experiment showed that there was no effect of self-touch on SEPs when the body parts in contact were not symmetric. Altogether, our findings suggest the interpretation that the secondary somatosensory cortex might underlie the specific analgesic effect of self-touch.

## Introduction

We experience a unitary bodily self-consciousness, despite the fact that the brain obtains information about the state and position of our body from a wide range of sensory sources. Even within the somatic modality, different inputs need to be integrated into this unitary experience, including information about muscle tone, tendon tension, and tactile input from the skin. Self-touch is a uniquely informative source of information about the body, since a person touching his or her own body is both the subject and the object of that contact, enforcing coherence between different somatic inputs. Accordingly, previous work has shown that self-touch plays a very important role in resolving conflict amongst conflicting somatic afferents (e.g. Lackner 1988). However, how self-touch affects the coherence of different somatosensory processes (tactile, proprioceptive, nociceptive, etc.) at a neural level is not understood.

It is a well-known phenomenon that pain resulting from afferent nociceptive input can be reduced by simultaneous tactile input from the same area, an observation that has been attributed to pain gating: a phenomenon that results from very early neural interactions at the level of the spinal cord (e.g. Melzack and Wall 1965). However, the experience of pain can be broken down into different components (Melzack 1993), which are known to be modulated at multiple stages in the central nervous system and are highly susceptible to influence from cognitive factors including attention, emotional state, and previous experiences (Bushnell et al. 2013). Importantly, recent work has shown that specifically self-touch (contact between one’s own limbs) can further reduce the perceived intensity of a painful stimulus in the limbs that are touching each other (Kammers et al. 2010). This finding was interpreted as a consequence of an increase in the ‘coherence of cognitive body representations to which pain afferents project’, but the level of neural processing at which self-touch affects the experience of pain is unknown.

Here, we use SEPs to investigate how self-touch affects incoming somatosensory signals. Incoming somatosensory signals evoke a number of cortical EEG components. These include early (N25, P60, N80) components thought to underlie processing of stimulus properties (Allison et al. 1992), midlatency (P100, N140) components that have been hypothesized to be involved in transferring stimulus information to awareness (e.g. Dehaene and Naccache 2001), and later, less modality-specific components related to cognitive factors such as attention (e.g. P300). Of particular a priori interest for the present question are the midlatency P100 and N140 components. Schubert et al. (2006) showed that whereas earlier SEP components (P60, N80) seem stimulus driven and do not correlate with conscious awareness, the midlatency P100 and N140 components are selectively enhanced in trials where a stimulus is perceived, as compared to trials where the stimulus is not perceived (for otherwise identical stimuli). This finding is therefore in agreement with suggestions that the neural processes underlying these midlatency components are involved in realizing the conscious experience of the incoming signals (e.g. Dehaene and Naccache 2001). This correspondence to the perceptual experience, rather than the stimulus itself, suggests that these processes might also be involved in the modulation of pain through self-touch as first reported by Kammers et al. (2010).

In two experiments, we investigate how SEPs evoked by painful shocks on the fingers depend on the object that the hands are in contact with. In Experiment 1, participants’ hands were touching each other (self-touch), not in contact with anything (no touch), or in contact with either an inanimate external object (a book) or an animate external object (the experimenter’s hand). In Experiment 2, we investigated whether self-touch modulates SEPs also during asymmetric self-touch, using a configuration where one of the participant’s hands was in contact with his or her own arm, whilst the other was in contact with the experimenter’s arm.

## Experiment 1

### Methods

#### Participants

Sixteen participants took part in the experiment after giving informed consent. All participants were right-handed and had normal or corrected-to-normal vision. The experiment was approved by the local ethics committee.

#### Setup

**Fig. 1:**
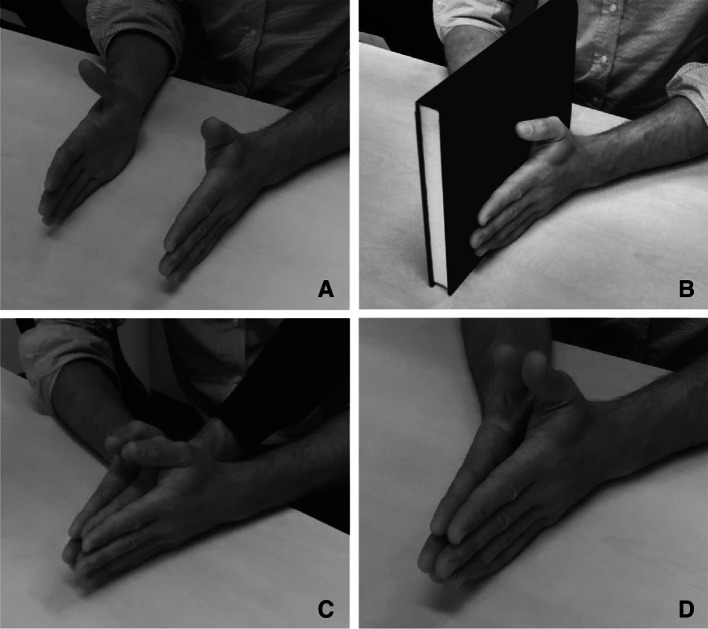
Experimental Setup. In all conditions, the participant’s two hands were resting on the table, thumbs upwards. In the no-touch condition (**a**), the hands were not in contact (the distance is exaggerated in the photograph—the real distance was approximately 10 cm). In the object-touch condition (**b**), the two hands were in contact with an inanimate object (a *hardcover book*). In the experimenter-touch condition (**c**), the two hands were in contact with an animate, external object (the experimenter’s *right hand*). Finally, in the self-touch condition (**d**), the participant’s two hands were pressed lightly together. For clarity, stimulation electrodes are not attached in this figure

Participants were seated at a table and fitted with a 16-channel Biosemi EEG cap. Stimulation electrodes were fitted to either side of the most distal phalanx of each middle finger, with the anode on the radial side and the cathode on the ulnar side. Participants rested their hands roughly 10 cm apart on the table, with their palms facing each other, thumb upwards, on either side of their vertical body midline. In four experimental conditions, the hands were either positioned apart as described (no touch), pressed lightly together (self-touch), pressed lightly together with a ~2 cm hardcover book in between their hands (object touch), or pressed lightly together with the experimenter’s right hand in between the two hands (experimenter touch). See Fig. 1 for photographs of each condition.

#### Apparatus

Shocks were administered using a custom-built Stanmore constant current stimulator (Stanmore, UK) controlled by a PC running Matlab 7.0 (The Mathworks, Natick, MA). Perceived shock strength depends on the total charge delivered to the electrodes and so depends on both current and pulse duration. In this experiment, current was kept constant at 40 mA and pulse duration was adjusted to adjust shock intensity.

#### Procedure

The experiment consisted of three blocks, each of which consisted of 20 trials. Over the course of each trial, a total of 80 shocks were administered approximately every 500 ms. Shock timing was jittered by adjusting each administration time by a value drawn from a uniform distribution of 0–50 ms. To ensure participants paid attention to the shocks during the experiment, they were required to detect target shocks which were slightly stronger than the baseline shocks. Participants used a foot pedal to indicate target shocks and were instructed to respond as rapidly as possible. Between zero and four target shocks could be administered per trial.

Within each block, the order of conditions was randomized with two restrictions. Firstly, each condition was presented once in the first four trials, once in the second four trials, once in the third four trials, and so on. In this way, we avoided that long-term adaptation effects might by chance affect one condition more than another. Secondly, consecutive trials were never the same condition, in order to avoid short-term adaptation and possible differences that might result from not needing to change between conditions. Within each trial, shocks were randomly administered to the left and right fingers.

Before the experiment, baseline shock duration was adjusted for each participant, independently for each of the two stimulated fingers, such that the baseline shocks were perceived as equal magnitude in both fingers and that they were experienced as just slightly painful. These shocks were presented repeatedly during the experiment and used to evoke the SEP. Likewise, before the first block, the amplitude of the target shocks was adjusted for each participant using a staircase procedure, such that the participant correctly detected 75 % of target shocks when presented amongst baseline shocks. To combat adaptation effects over the course of the experimental session, these matching procedures were repeated between each of the three experimental blocks and pulse durations were adjusted if necessary. Across participants and over the course of the entire experiment, mean pulse durations of standard shocks were 35.5 µs (SD 10.5 µs) for the left hand and 39.2 µs (SD 9.6 µs) for the right hand. Mean durations for target shocks were 52.9 µs (SD 15.7 µs) for the left hand and 64.6 µs (SD 20.4 µs) for the right hand.

#### EEG analysis

Sixteen-channel EEG recordings were acquired at 2048 Hz from recording sites Fz, F3, F4, Cz, C3, C5, C4, C6, Pz, P3, P5, P4, P6, Oz, O1, and O2 using a 16-channel Biosemi EEG amplifier and ActiView software (Biosemi, Amsterdam, The Netherlands) and analysed using Matlab 7.0 (The Mathworks, Natick, MA) with EEGlab 8 extensions (Delorme and Makeig 2004). Recordings were referenced to the mean of the left and right mastoid. Data were resampled offline to 512 Hz and epochs were extracted from −250 to 1000 ms relative to the shock stimulus. Epoch baselines were set to the mean of 100 ms preceding the stimulus. Target shocks and shocks administered within 2 s of either a target stimulus or the participant’s response were excluded from analysis.

Simultaneously recorded vertical electrooculogram (VEOG) data were inspected for eye movement artefacts using an automated procedure. Trials with any timepoint exceeding 75 µV amplitude on any EEG channel, or greater than 200 µV difference between VEOG channels at any timepoint, were excluded from further analysis. We applied this relatively strict inclusion criterion because participants were required to move between conditions and were also in contact with another person in some conditions, yielding a lot of eye movement and muscle artefacts. This strict inclusion criterion resulted in the rejection of a substantial portion of the recorded data; participants for whom this procedure resulted in greater than 60 % of trials being removed were omitted from further analysis (corresponding to fewer than 160 trials per condition on average). Four participants were removed in this way, leaving 12 participants in the final analysis. We chose to apply to this conservative rejection procedure, even though it resulted in the rejection of a substantial portion of our dataset, because it reduced the chance of generating false-positive results caused by artefacts that systematically differ between conditions.

### Results

Following Schubert et al. (2006), we were especially interested in the somatosensory P100 and N140, since these two components seem likely early candidates for the realization of conscious awareness of incoming stimuli. However, we also investigated three earlier SEP components in order to rule out possible earlier interactions: the N25, P60, and N80. These components are thought to reflect initial cortical processing of afferent input to primary somatosensory cortex SI and are therefore defined over contralateral C3/4. We defined N25 amplitude as the mean amplitude from 10 to 40 ms, P60 amplitude as the mean amplitude from 40 to 70 ms, and N80 amplitude as the mean amplitude from 60 to 90 ms. Each component was submitted to a repeated measures analysis of variance. None of the comparisons were found to be significant (all *p* > 0.63, uncorrected).

#### P100

The P100 component is the earliest somatosensory evoked potential that has been shown to be modulated by cognitive factors such as task demands (e.g. Desmedt et al. 1983) and to correlate with conscious awareness (e.g. Schubert et al. 2006). The bilateral central scalp distribution of this component suggests an origin in bilateral secondary somatosensory cortices (SII), in line with magnetoencephalography work showing that both median and ulnar nerve stimulation evoke event-related fields that are likely to originate from contralateral activation of primary somatosensory cortex (SI) peaking earlier at 35 ms, followed by bilateral activation of SII around 90 ms poststimulus (Forss et al. 1996).

**Fig. 2:**
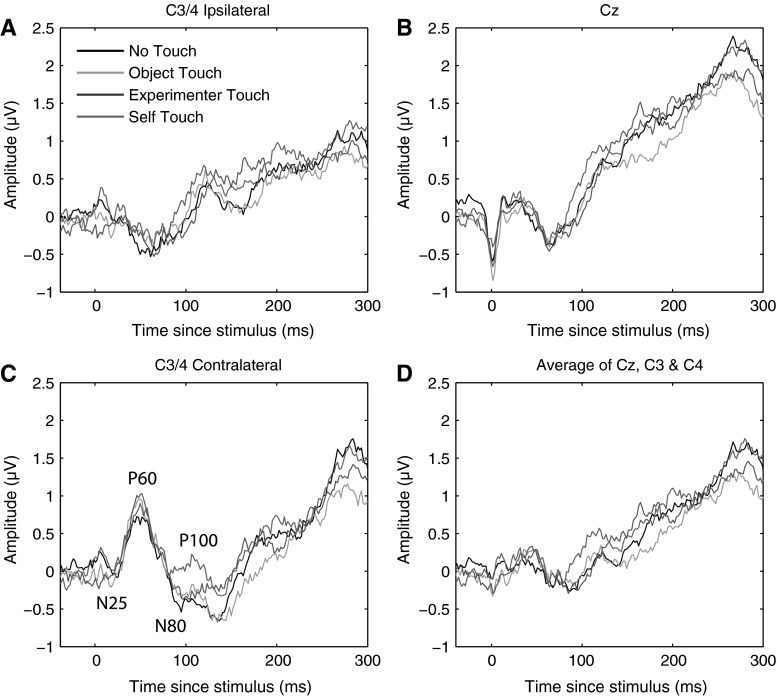
Grand average somatosensory evoked potentials for all four conditions at C3/4 ipsilateral, Cz, C3/4 contralateral, and all three electrode locations averaged together. A positive deflection around 80–110 ms of the self-touch condition with respect to the other conditions is evident at all electrodes individually as well as in the average

**Fig. 3:**
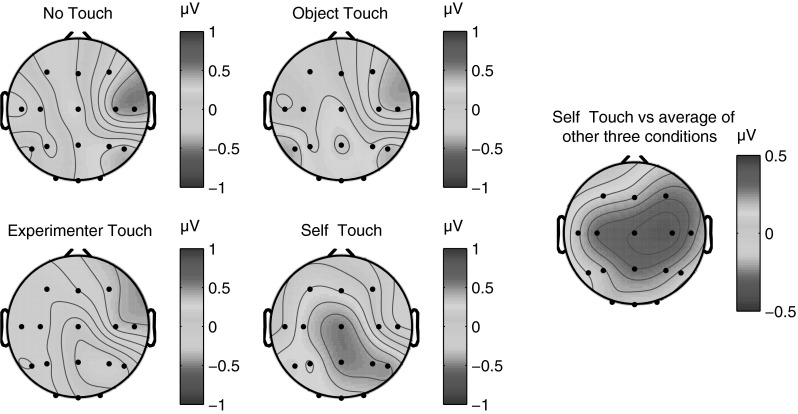
Grand average scalp distribution of P100 for all four conditions, as well as the difference scalp map of self-touch compared to the average of the other three conditions (average amplitudes over 80–110 ms in all cases). Scalp distributions of right-hand shocks are mirrored such that these scalp distributions show ipsilateral activity on the *left* and contralateral activity on the *right*

We defined the P100 component as the mean ERP amplitude recorded over recording sites Cz, C3, and C4 from 80 to 110 ms poststimulus. Averaging across experimental conditions, the P100 component could be isolated as a local positive deflection in the overall waveform in 10 out of 12 individual participants. Figure 2 below shows electrode traces for each of these electrodes individually for each of the four experimental conditions, as well as for the three electrodes averaged together. Figure 3 further shows scalp distributions of the mean ERP amplitude over the 80–110 ms period for each of the four conditions, as well as a difference map of self-touch versus the other three conditions.

**Fig. 4:**
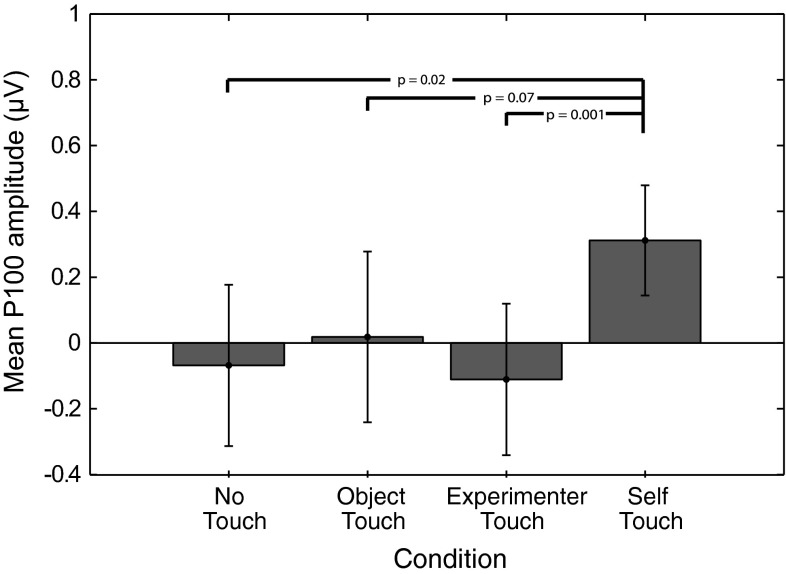
Mean P100 amplitude in each of the four experimental conditions. *Error bars* represent standard errors of the mean across 12 participants. A one-way ANOVA revealed a significant effect of condition, which is driven by an increase in amplitude in the self-touch condition

P100 amplitude was analysed by averaging amplitudes recorded at Cz, C3, and C4 over all timepoints from 80 to 110 ms poststimulus, and the resulting average amplitudes were submitted to a repeated measures analysis of variance. This revealed a main effect of condition (*F* = 3.549, *df* = 3, *p* = 0.025). Follow-up paired-samples *t* tests revealed that this effect is driven by an increase in P100 amplitude in the self-touch condition (self vs none: *t* = −2.7, *df* = 11, *p* = 0.02; self vs object: *t* = −1.992, *df* = 11, *p* = 0.07; self vs experimenter *t* = −4.238, *df* = 11, *p* = 0.001); see Fig. 4. After Holm–Bonferroni correction for three simultaneous comparisons, the self-touch condition differed significantly from the experimenter-touch and the no-touch conditions.

#### N140

**Fig. 5:**
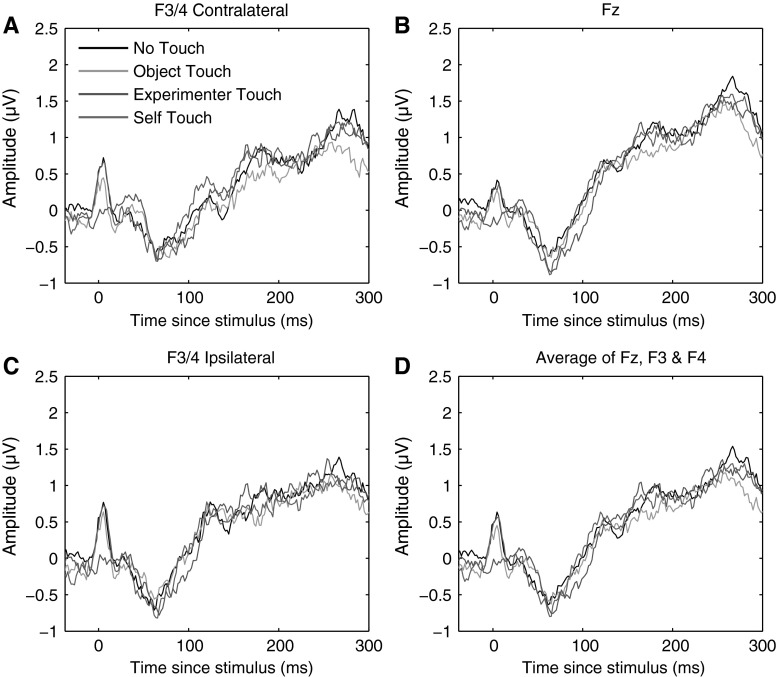
Grand average somatosensory evoked potentials for all four conditions at F3/4 ipsilateral, Fz, F3/4 contralateral, and all three electrode locations averaged together. No systematic differences between conditions were evident at any of these electrodes in any time frame

**Fig. 6:**
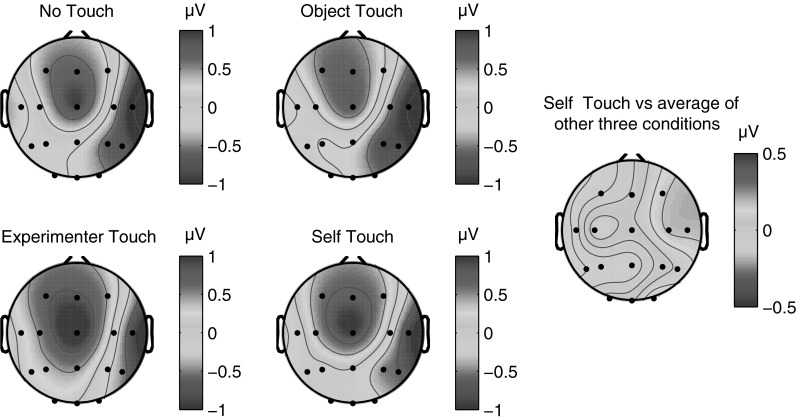
Grand average scalp distribution of N140 for all four conditions, as well as the difference scalp map of self-touch compared to the average of the other three conditions (average amplitudes over 130–160 ms in all cases). Scalp distributions of right-hand shocks are mirrored such that these scalp distributions show ipsilateral activity on the *left* and contralateral activity on the *right*

The N140 somatosensory evoked component traditionally has a bilateral frontal distribution and peaks between 130 and 160 ms poststimulus (Michie et al. 1987). Like the P100 component, N140 amplitude is also correlated with conscious awareness of the stimulus (Schubert et al. 2006). We defined the N140 component as the mean ERP amplitude recorded over recording sites Fz, F3, and F4, from 130 to 160 ms poststimulus. Figure 5 below shows electrode traces for each of these electrodes individually for each of the four experimental conditions, as well as for the three electrodes averaged together. Figure 5 further shows scalp distributions of mean ERP amplitudes over the 130–160 ms period for each of the four conditions, as well as a difference map of self-touch versus the other three conditions (Fig. 6).

**Fig. 7:**
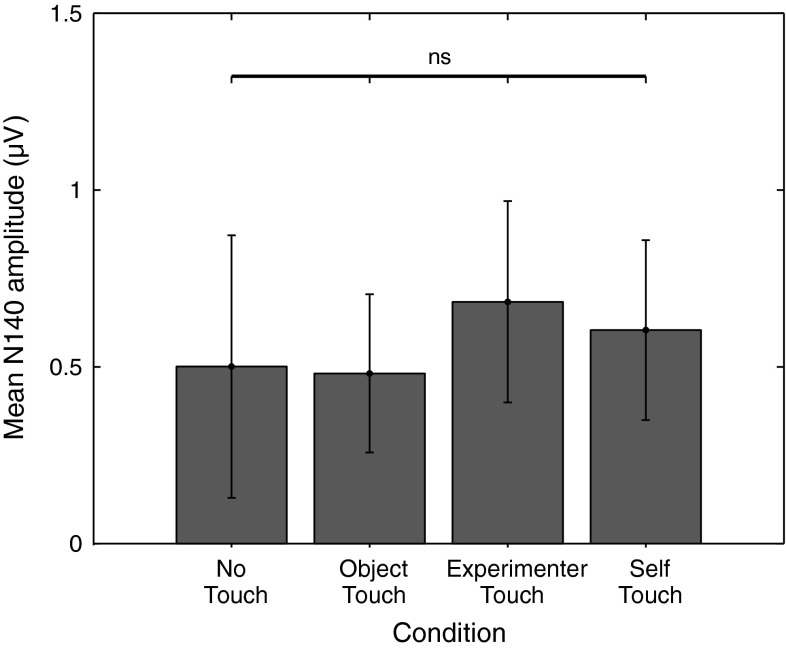
Mean N140 amplitude in each of the four experimental conditions. *Error bars* represent standard errors of the mean across 12 participants. No significant differences in N140 amplitude between conditions were observed

N140 amplitude was analysed by averaging amplitudes recorded at Fz, F3, and F4 over all timepoints from 130 to 160 ms poststimulus, and the resulting average amplitudes were submitted to a repeated measures analysis of variance. This revealed no main effect of condition (*F* = 0.474, *df* = 3, *p* = 0.70); see Fig. 7 for average N140 amplitudes for each condition.

#### Behaviour

**Fig. 8:**
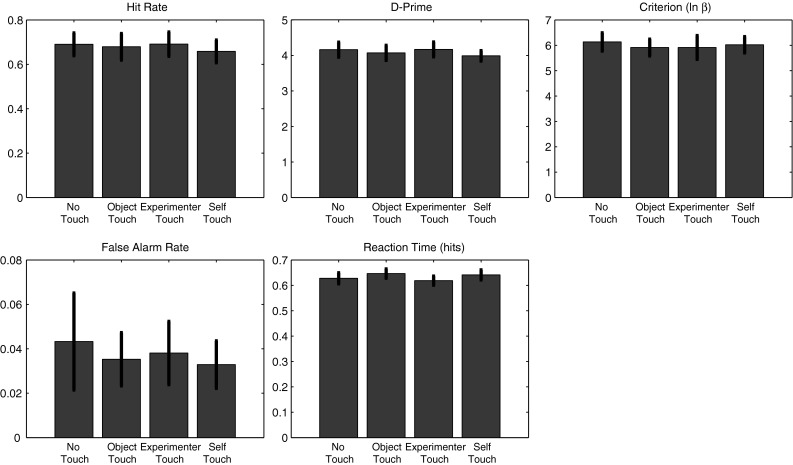
Experiment 1 behavioural results. There were no significant differences between conditions for any of the following measures: hit rate, false-alarm rate, *d*-prime, criterion, or reaction time

Participant responses were analysed in a signal detection framework. Each of the measures hit rate, false-alarm rate, *d*-prime, criterion (ln(*β*)), and reaction time was subjected to a repeated measures analysis of variance across all 12 participants. There were no significant effects of condition on any of the variables tested (all *p* > 0.09). Figure 8 shows all measures for all conditions.

### Interim conclusion

SEPs evoked by a shock on either of the middle fingers were recorded whilst the participant’s two hands were either not touching anything, touching an inanimate object, touching an external animate object (the experimenter’s hand), or touching each other (self-touch). We observed a significant increase in the amplitude of the P100 SEP component over bilateral central electrode sites in the self-touch condition as compared to the other three conditions. Conversely, we observed no differences between conditions for the N140 SEP component over frontal electrode sites. Behavioural measures showed no differences between any of the conditions.

The significant increase in P100 amplitude for the self-touch condition as compared to the experimenter-touch and no-touch conditions suggests a possible role for this component in underlying the analgesic effect of self-touch as reported by Kammers et al. (2010). Furthermore, the bilateral central scalp distribution of the difference wave suggests a locus in the secondary somatosensory cortex (SII), which is known to be active at this latency (Forss et al. 1996). The notion that the modulating effect of self-touch might be localized to SII seems particularly plausible since self-touch between two opposite limbs (the two hands, in this case) inevitably generates correlated input to the two hemispheres, which SII is likely to be sensitive to given its bilateral response to both painful and non-painful stimuli (e.g. Feretti et al. 2003). As per Hebbian learning, this correlated activity might temporarily increase functional connectivity between the two areas and consequently affect the processing of further incoming stimuli. Although Hebbian learning is traditionally used to explain long-term connectivity changes such as long-term potentiation, it seems plausible that the continuous correlating input resulting from sustained contact between opposite limbs might also have more short-term effects. Furthermore, receptive fields of SII neurons in macaques are not only bilateral, but also show complex tuning properties, consistent with interhemispheric transfer at a relatively high level of hierarchical processing (Iwamura et al. 1994).

Bilateral SII neurons show somatotopically symmetric receptive field properties (Feretti et al. 2003). Therefore, if SII neurons with bilateral receptive fields are the underlying neural source of the P100 modulation observed in Experiment 1, this would predict that the effect of self-touch on the P100 SEP component should disappear if self-touch would be asymmetric. In the next experiment, we test this prediction.

## Experiment 2

**Fig. 9:**
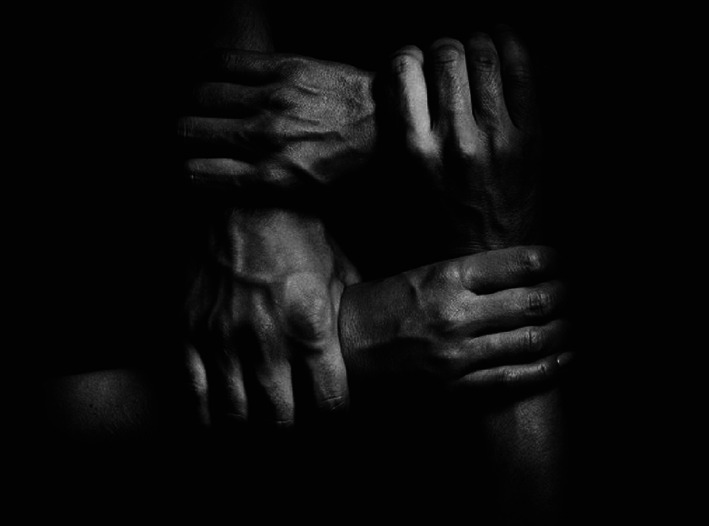
Fireman’s chair configuration

In Experiment 2, shocks were administered to participants’ hands whilst participants were seated across from an experimenter, grasping each other’s hands in a ‘fireman’s chair’ (Fig. 9). In this configuration, one hand was always touching the experimenter whilst the other was touching the participant’s own body. In this way, we aimed to investigate whether self-touch also affects the midlatency SEP components when the body parts in contact are asymmetric.

### Methods

#### Participants

Eleven right-handed participants with normal or corrected-to-normal vision took part in the experiment after giving informed consent. The experiment was approved by the local ethics committee.

#### Apparatus

Shocks were administered to self-adhesive stimulation electrodes using two Digitimer DS7A constant current stimulators (Digitimer Ltd, UK) controlled by a PC running Matlab 7.0 (The Mathworks, Natick, MA). A different stimulus device was used in Experiment 2 because the device used in Experiment 1 proved unreliable at generating simultaneous shocks on multiple channels. Perceived shock strength was adjusted during the experiment by keeping the current constant at 20 mA whilst adjusting the pulse duration. Technical limitations of the device prevented us from using 40 mA constant current as in Experiment 1. However, pilot experiments revealed that stimuli from either device that were matched for total charge (current × duration) were indistinguishable.

#### Setup

The participant was seated opposite the experimenter, with the left arm extended and the right elbow flexed such that the right hand grasped the left wrist. The experimenter did the same, and the experimenter and participant used their free hands to grasp the other’s wrist in a ‘fireman’s chair’ configuration (Fig. 9). In half of the blocks, the configuration was mirrored, switching which of the two hands was in contact with the experimenter and which was in contact with the participant himself/herself. Together, these conditions dissociate the identity of the person that each hand touches whilst providing comparable somatosensory input (the glabrous dorsal side of the wrist in all cases). Furthermore, the total physical contact between the two bodies remained constant across the two conditions. This setup allowed us to directly compare SEPs evoked during local experimenter-touch and local self-touch conditions by simply contrasting the two hands.

#### Procedure

Participants took part in 20 experimental trials in a single session. Participants started randomly with either their left or right hand grasping the experimenter, after which they switched positions between each block. During each trial, 80 shocks were randomly administered to the most distal phalanx of the middle fingers of each of the two hands. However, whereas in Experiment 1 participants monitored the train of shocks for a higher-amplitude target, in Experiment 2 participants monitored the train of individual shocks for two identical shocks administered simultaneously to each of the two hands. Participants reported targets using a foot pedal. The only purpose of the task was to ensure participants attended to both hands equally; behavioural measures were not analysed other than to verify that all participants correctly detected at least 50 % of target shocks. Between two and five targets were presented per block. Target shocks and the first two normal shocks that followed a target were removed from further analysis to avoid contaminating the EEG with activity related to target detection or motor preparation.

The setup and procedure were otherwise comparable to the previous experiment. As in Experiment 1, 16-channel EEG was acquired using a 16-channel Biosemi EEG amplifier and ActiView software (Biosemi, Amsterdam, the Netherlands) and analysed using Matlab 7.0 (The Mathworks, Natick, MA) with EEGlab 8 extensions (Delorme and Makeig 2004). Preprocessing was identical to Experiment 1. Two participants were removed from further analysis due to fewer than 40 % of valid trials remaining following automatic artefact rejection, leaving nine participants in the final analysis.

### Results

As in Experiment 1, we were interested in the P100 and N140 SEP components due to their putative role in generating conscious awareness. However, we also tested earlier components (N25, P60, N80) as in Experiment 1. Using paired-samples *t* tests to compare conditions, none of these components revealed a significant difference between conditions (all *p* > 0.39).

#### P100

**Fig. 10:**
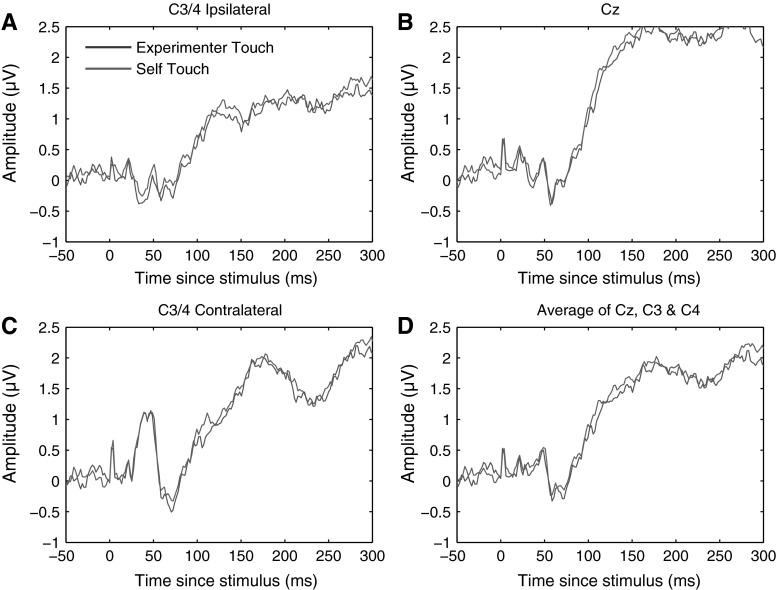
Grand average somatosensory evoked potentials for both conditions at C3/4 ipsilateral, Cz, C3/4 contralateral, and all three electrode locations averaged together. No systematic differences between experimenter-touch and self-touch conditions were evident at any timepoint

**Fig. 11:**
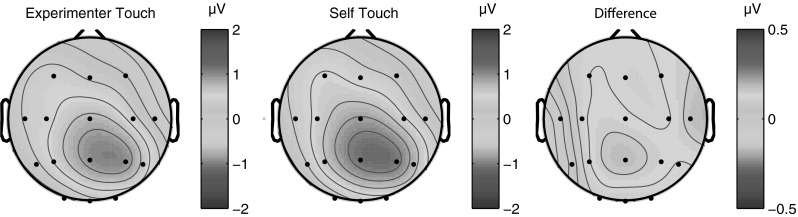
Grand average scalp distribution of P100 for both experimenter-touch and self-touch conditions, as well as the difference map between conditions (averaged from 80 to 110 ms in all cases). Scalp distributions of right-hand shocks are mirrored such that these scalp distributions show ipsilateral activity on the *left* and contralateral activity on the *right*

We defined the P100 component in the identical way to Experiment 1 as the mean ERP amplitude recorded over recording sites Cz, C3, and C4 from 80 to 110 ms poststimulus. Averaging across experimental conditions, the P100 component could be isolated as a local positive deflection in the overall waveform in eight out of nine individual participants. Figure 10 shows electrode traces for each of these electrodes individually for both experimental conditions, as well as for all three sites averaged together. Scalp distributions over the 80–110 ms period are shown in Fig. 11.

P100 amplitude was analysed in the same way as for Experiment 1, by averaging amplitudes recorded at Cz, C3, and C4 over all timepoints from 80 to 110 ms poststimulus. The resulting average amplitudes did not significantly differ between conditions (paired-samples *t* test; *t* = −1.17, *df* = 8, *p* = 0.27).

#### N140

**Fig. 12:**
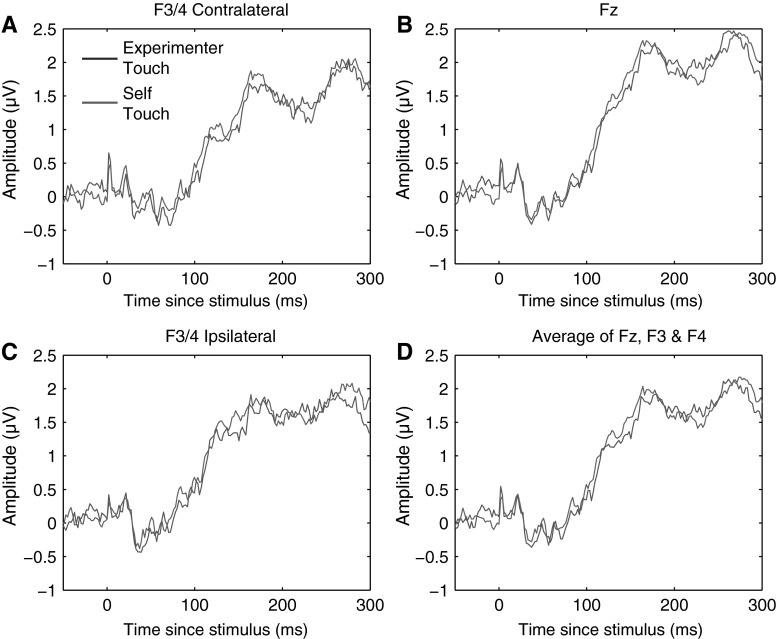
Grand average somatosensory evoked potentials for both experimental conditions at F3/4 ipsilateral, Fz, F3/4 contralateral, and all three electrode locations averaged together. No systematic differences between experimenter-touch and self-touch conditions were evident at any timepoint

**Fig. 13:**
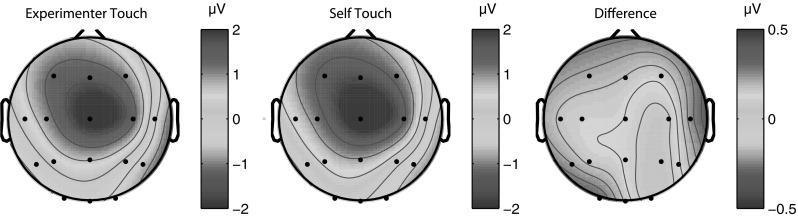
Scalp distribution of N140 for both experimenter-touch and self-touch conditions, as well as the difference map (averaged from 130 to 160 ms in all cases). Scalp distributions of right-shock trials have been mirrored so that all scalp maps show ipsilateral activation on the *left* and contralateral activation on the *right*

We analysed the N140 SEP component in the same way as for Experiment 1, defining N140 amplitude as the mean amplitude recorded over recording sites Fz, F3, and F4 (both ipsilateral and contralateral to the stimulation side), averaged from 130 to 160 ms poststimulus. Figure 12 below shows electrode traces for each of these electrodes individually, as well as for the three electrodes averaged together. Figure 13 further shows average scalp distributions for each condition, as well as a difference map of self-touch versus experimenter touch. A paired-samples *t* test revealed that average N140 amplitude did not significantly differ between conditions (*t* = −0.93, *df* = 8, *p* = 0.37).

#### Further analysis

Applying the identical analysis approach to Experiment 2 that was applied to Experiment 1 yielded no significant differences in either P100 amplitude or N140 amplitude between local experimenter touch and local self-touch. However, in order to further explore the data for any indication that local somatosensory input is differently processed during self-touch versus experimenter touch, we subsequently analysed the data using multivariate pattern classification. Commonly applied to fMRI data, this has also been shown to be an effective technique for MEG (e.g. Carlson et al. 2011, 2013) and EEG data (Hogendoorn et al. 2015). This technique incorporates any systematic information available at any electrode at a given timepoint, without a priori assumptions about location, polarity, or timepoint. For each timepoint in an EEG epoch, a multivariate linear discriminant classifier is trained to distinguish two kinds of trials on the basis of the pattern of electrode potentials. The classifier is trained using half of all individual EEG trials and tested on the remaining individual trials, after which training and test trials are switched and the analysis is repeated. Any above-chance classifier performance indicates the availability of systematic differences in the EEG between the two conditions at that timepoint.

**Fig. 14:**
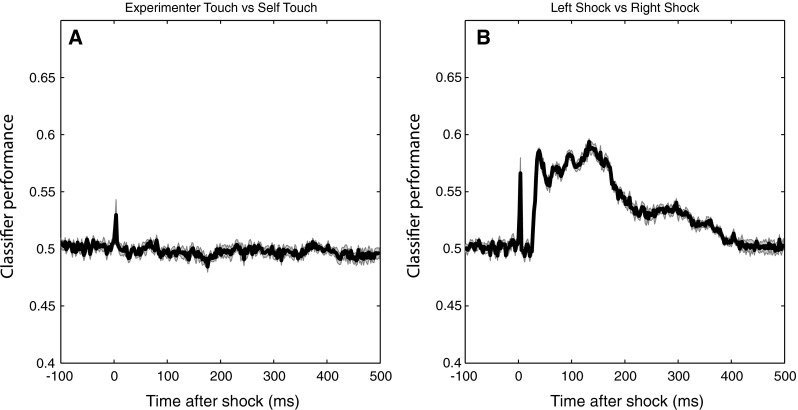
Performance over the course of the epoch of a linear discriminant classifier trained to distinguish individual EEG epochs. The classifier in (**a)** was trained to discriminate shocks during experimenter touch versus self-touch, and the classifier in (**b)** was trained to discriminate left-hand shocks versus right-hand shocks. Chance performance in both cases is 0.5, and *shaded areas* indicate standard errors of the mean. Any above-chance classification performance indicates the availability of systematic information in the EEG at that timepoint discriminating the respective categories. The classifier is unable to distinguish self-touch from experimenter touch (**a**) but performs above chance when distinguishing left-hand from right-hand shocks, as expected (**b**). The spikes in classification performance at *t* = 0 in both panels reflect the classifier’s above-chance ability to discriminate whether the stimulus artefact was at either a self-touch or experimenter-touch location (**a**) or on the left or right middle finger (**b**)

Here, the classifier was trained to discriminate self-touch and experimenter-touch trials for the left and right hands separately. As shown in Fig. 14a, classification performance remains at chance throughout the entire epoch. A brief spike in classification performance at 0 ms indicates that this analysis approach is sensitive enough to discriminate how the stimulus artefact itself is modulated by whether the stimulated hand is contacting the participant’s own body or the experimenter’s.

To further demonstrate that the classifier is able to extract systematic information from the EEG at all, a new set of classifiers was trained to discriminate left-shock and right-shock trials. Figure 14b shows that a classifier is easily able to discriminate the laterality of the shock, starting with the very first cortical activation at 30 ms after the stimulus (as well as being able to discriminate the laterality of the stimulus artefact itself). This sensitivity of the classifier technique makes it unlikely that the null result comparing experimenter touch to self-touch is due to insufficient power.

## Overall discussion

Kammers et al. (2010) reported an analgesic effect of self-touch, over and above the well-known reduction in pain that results from concurrent tactile input from overlapping receptive fields (pain gating; Melzack and Wall 1965). The current study aimed to shed light on the neural mechanisms underlying this additional modulatory effect of self-touch. In two experiments, we investigated the effect of self-touch on two midlatency somatosensory evoked components (P100 and N140). Experiment 1 showed that the amplitude of the P100 component evoked by a shock is significantly increased when the participant is touching his or her hands together, as compared to when he or she is not touching anything, touching an inanimate object, or touching the experimenter’s hand. The N140 component was not affected.

In Experiment 2, we investigated whether this modulatory effect of self-touch was contingent on symmetric contact between body parts, as would be predicted by a putative neural source at the level of the secondary somatosensory cortex SII. Participants simultaneously touched themselves with one hand and touched the experimenter with the other, and we found that SEPs evoked by shocks on the self-touch hand did not differ from SEPs evoked by shocks on the hand touching the experimenter. To eliminate the possibility that this null result was due to a lack of power, we reanalysed the data using a multivariate pattern classification approach and again found no difference between self-touch and experimenter touch when both were present simultaneously on different hands, suggesting that somatosensory input was processed equivalently in the two cases.

In Experiment 1, we did not observe any effect of self-touch on behavioural performance (i.e. the ability of the participants to discriminate baseline shocks from slightly stronger target shocks). On the basis of finding reported by Kammers et al. (2010), one might expect behavioural performance to suffer in the self-touch condition due to the reduction in perceived intensity of the shock stimulus. However, target shocks were presented as part of a train of non-target shocks that would similarly be attenuated. Furthermore, pain is a multidimensional experience (Melzack 1993) whereby an effect on the motivational-affective dimension (i.e. the unpleasantness of a stimulus) need not necessarily generalize to its sensory-discriminative properties.

Experiment 1 suggests that the midlatency somatosensory evoked potential P100 is potentially involved in the modulation of afferent pain signals by self-touch. The bilateral central scalp distribution of the difference wave (Fig. 3), together with its timing, suggests that the effect might originate in bilateral secondary somatosensory cortices (SII). In the present experiments, we acquired only 16-channel EEG data, limiting the degree to which we can localize the source of the effect. Nevertheless, this interpretation is consistent with SII’s known early bilateral response characteristics (e.g. Forss et al. 1996; Iwamura et al. 1994) as well as the fact that the tuning properties of SII neurons suggest that they integrate proprioceptive and tactile information (Fitzgerald et al. 2006a, b). Disbrow et al. (2001) showed that stimulation of the hands does indeed result in ipsilateral processing in SII and that SII in each hemisphere processes bimanual input, whereby ipsilateral input modulates simultaneous incoming contralateral input. Because bilateral neurons in SII show somatotopically symmetric receptive fields (Feretti et al. 2003), such an interpretation predicts that self-touch between symmetric body parts would be required for self-touch to affect neural processing at this latency. We tested this prediction in Experiment 2, which indeed showed that the P100 SEP component did not differ between local self-touch and local experimenter touch when self-touch was asymmetric.

We therefore believe that SII is well situated in the somatosensory processing hierarchy to both detect self-touch and modulate ascending input. However, it remains an open question whether the mechanisms detecting self-touch require symmetric contact of the hands specifically or whether similar effects might be found for contact between any symmetric body parts. Bimanual coordination is clearly an evolutionarily important ability, and less is known about bilateral processing of input from other, non-hand areas or whether inputs from two non-symmetric limbs (e.g. left hand with right foot) interact in similar ways. As such, it remains an open question whether self-touch has similar effects when it involves symmetric non-hand body parts.

The idea that self-touch might first be detected in SII is also partly consistent with findings from SEP studies using different experimental paradigms. For instance, Aspell et al. (2012) found that during illusions of full-body localization, midlatency SEPs evoked by tibial nerve stimulation were modulated when the illusion was induced by synchronous stroking as compared to a control condition with asynchronous stroking. However, there are also clear differences between SEPs evoked during body-ownership illusions and the effects of self-touch. For instance, Aspell and colleagues also found effects on an early SEP component, which we did not. Likewise, Dieguez et al. (2009) reported modulation of SEPs thought to originate in SI, rather than SII, following median nerve stimulation during a finger-ownership illusion. As such, a comparison between the underlying mechanisms of self-touch and body-ownership remains speculative.

We did not acquire subjective pain ratings from participants for two reasons. Firstly, stimulus amplitudes were adjusted for each participant individually at the start of the experiment such that they were perceived as just barely painful, in order to ensure activation of nociceptive pathways without making it unbearable to sit through 4800 shocks over the course of the experiment. Furthermore, we deliberately adjusted shock amplitudes during the experiment to keep the subjective experience as constant as possible. As such, we anticipated very little reliable variance in subjective intensity reports. Secondly, because the subjective experience of pain (and so potentially the processing of nociceptive afferents) is susceptible to higher-order influences, we did not want to draw attention to pain as a variable in this experiment. This avoided possible confounds arising from expectations or other cognitive effects. Nevertheless, this limits the degree to which the present conclusions can be directly linked to the actual experience of pain. In the present experiments, we deliberately applied relatively powerful shocks, in order to stimulate all afferent fibre channels. Classically, nociceptive afferents are carried on a-delta and c-fibres, with a-beta fibres carrying innocuous tactile input. For instance, Mouraux et al. (2010) found that both electrophysiological and behavioural responses to intra-epidermal nociceptive stimulation were abolished following denervation of capsaicin-sensitive a-delta afferents. However, Djouhri and Laweson (2004) showed that in many species, this dichotomy is far from absolute, with many nociceptors actually projecting via a-beta fibres. In any case, it remains an open question which pathways are involved in the modulation of somatosensory afferents during self-touch.

What would be the evolutionary advantage of self-touch producing analgesia? It is well established that pain ratings for both acute and chronic pain can be modulated by higher-level cognition (e.g. Longo et al. 2009; Mancini et al. 2011; Moseley et al. 2008). One such cognitive factor is the perceived threat of the painful stimulus (for an overview, see Butler and Moseley 2003). Whereas being touched by someone else is inherently uncertain, self-touch reduces such uncertainty and provides predictability and possibly a sense of protection or safety. Conversely, contact with another person is unpredictable and could thereby be perceived as a liability, especially when we are in pain.

In Experiment 2, we observed that asymmetric self-touch did not differentially affect the P100 SEP component as compared to touching the experimenter. Although this pattern of results fits well with the known bilateral symmetric response properties of SII neurons, P100 scalp distributions for both conditions in Experiment 2 (Fig. 11) show similarities to the self-touch condition in Experiment 1 (Fig. 3). The fact that the scalp distribution of the experimenter-touch condition in Experiment 2 appears more similar to the self-touch (as opposed to experimenter touch) condition of Experiment 1 hints that perhaps some P100 modulation might in fact also be taking place in both conditions in Experiment 2. Although it is speculative, such modulation might reflect that the fireman’s chair configuration is a very stable, interlinked configuration generating correlated and predictable input to both limbs, irrespective of the identity of the limbs being touched by each hand.

Clearly, more research will be necessary to investigate whether the predictability of touch might affect processing of afferent input. Indeed, such an interpretation might predict that removing the predictability of self-touch (for example if the person’s limbs are passively brought into contact with one another), this might reduce the analgesic effect of self-touch. Conversely, this interpretation would predict that increasing the predictability of external touch might alleviate pain. Whether this occurs is currently an open empirical question, but if uncertainty and predictability of touch do underlie this aspect of the pain experience, this would have clinical implications for the interaction with patients who are in pain, where it would therefore be good practice that the doctor talks the patient through a procedure with the aim of reducing uncertainty about when and where the patient will be touched.

In sum, in this study, we followed up on previous reports of additional analgesia resulting from self-touch by investigating SEPs evoked by painful stimuli. We show that the midlatency P100, which has previously been implicated in the conscious perception of a sensory stimulus, is enhanced when the painful stimulus is administered whilst the person is touching one’s own body, as compared to when he or she is touching nothing, an inanimate object, or another person’s hand. A follow-up experiment showed that the effects of self-touch on SEPs disappear when contact between body parts is asymmetric, suggesting that the underlying mechanisms operate at a level where receptive fields are large enough to encompass both of the hands. Based on the scalp distribution of the P100 difference wave, its timing, and its position in the somatosensory processing hierarchy as the earliest processing area with bilateral response characteristics, we suggest that secondary somatosensory cortex might underlie this effect.
